# Microconvex Probe-Assisted Sonopalpation for Ultrasound-Guided Diagnosis of Cervical Discogenic Pain in Challenging Neck Anatomy: A Technical Report

**DOI:** 10.7759/cureus.114238

**Published:** 2026-08-09

**Authors:** King Hei Stanley Lam, Yonghyun Yoon, Teinny Suryadi, Anwar Suhaimi, Daniel Chiung-Jui Su

**Affiliations:** 1 Clinical Research, International Academy of Regenerative Medicine, Incheon, KOR; 2 The Board of Clinical Research, The Indonesian Malaysian Regenerative Institute, Jakarta, IDN; 3 The Board of Clinical Research, The International Association of Musculoskeletal Medicine, Kowloon, HKG; 4 Faculty of Medicine, The Chinese University of Hong Kong, New Territories, HKG; 5 Faculty of Medicine, The University of Hong Kong, Hong Kong, HKG; 6 The Board of Clinical Research, The Hong Kong Institute of Musculoskeletal Medicine, Kowloon, HKG; 7 Orthopedics, International Academy of Musculoskeletal Medicine, Hongkong, HKG; 8 Orthopedics, International Academy of Regenerative Medicine, Incheon, KOR; 9 Orthopedic Surgery, Hallym University Gangnam Sacred Heart Hospital, Seoul, KOR; 10 Orthopedic Surgery, Incheon Terminal Orthopedic Surgery clinic, Incheon, KOR; 11 Physical Medicine and Rehabilitation, Medistra Hospital, Jakarta, IDN; 12 Physical Medicine and Rehabilitation, Synergy Clinic, Jakarta, IDN; 13 Physical Medicine and Rehabilitation, Hermina Podomoro Hospital, Jakarta, IDN; 14 Rehabilitation Medicine, University Malaya Medical Centre, Kuala Lumpur, MYS; 15 Rehabilitation Medicine, University Malaya, Kuala Lumpur, MYS; 16 Physical Medicine and Rehabilitation, Chi Mei Medical Center, Tainan, TWN

**Keywords:** anterior cervical approach, cervical discogenic pain, cervical ultrasound, difficult neck anatomy, disc sonopalpation, microconvex probe, prevertebral muscles, sonopalpation, technical report, ultrasound-guided diagnosis

## Abstract

Cervical discogenic pain is diagnostically challenging because degenerative changes on magnetic resonance imaging often do not reliably identify the symptomatic disc level. Although provocative discography remains an important reference standard in selected patients, it is invasive and controversial. More recently, ultrasound-guided sonopalpation has been described as a practical adjunct for identifying painful cervical discs before intradiscal intervention. However, in patients with short necks, bulky anterior soft tissues, reduced tissue compliance, or close apposition of the carotid sheath to the thyroid-tracheal complex, conventional finger-based sonopalpation may be technically difficult or impossible.

This technical report describes a focused modification of previously published methods: use of the vertex of a small-footprint microconvex transducer as the sonopalpation instrument itself. After standard ultrasound localization of the target cervical level, the probe is advanced into the sagittal plane between the carotid sheath and the thyroid-tracheal complex under real-time visualization. The curved apex of the transducer is then used to apply graded focal pressure through the longus colli and through the longus capitis when appropriate at more cephalad levels, to the anterolateral annulus fibrosus while the patient is asked whether concordant pain is reproduced. If intervention is planned, the same transducer may then be used for subsequent ultrasound-guided disc access according to previously described medial cervical approaches.

In our practical setup, the patient may be examined in the seated position while facing the ultrasound screen, with the operator standing behind the patient. This arrangement allows real-time patient feedback and may help reproduce the patient’s usual mechanical pain state through gravitational loading of the head and cervical spine during focused disc compression.

This modification extends prior ultrasound-guided cervical disc techniques by allowing the microconvex probe to function not only as an imaging device and tissue separator, but also as a direct palpation tool. Its potential advantages are improved access in difficult anatomy, simultaneous tissue displacement and symptom provocation, and continuous visualization of critical structures. The principal trade-off is reduced tactile feedback compared with digital palpation. Further clinical study is needed to define reproducibility, diagnostic value, and safety.

## Introduction

Neck pain is a common musculoskeletal complaint with substantial functional and socioeconomic impact worldwide [1]. Among its many causes, cervical intervertebral disc degeneration is an important but often difficult-to-confirm source of axial neck pain and referred shoulder or interscapular pain [2,3]. The concept of cervical discogenic pain is biologically plausible because degenerated cervical discs may develop nociceptive innervation, inflammatory activity, and mechanical sensitization that contribute to pain generation [2,3]. Cervical disorders may present with heterogeneous symptoms, but proving that a specific disc is the primary pain generator remains difficult.

A major diagnostic limitation is that structural abnormalities on magnetic resonance imaging (MRI) do not reliably identify the symptomatic disc level. Disc desiccation, annular fissuring, and multilevel degeneration are common, and the severity of imaging abnormalities may not correspond to the patient’s symptoms [4-6]. While MRI remains essential for structural assessment, it is a static study and cannot by itself determine which degenerated cervical level is mechanically symptomatic [4-6]. Classic correlation studies showed that cervical MRI can miss painful annular pathology and cannot consistently distinguish symptomatic from asymptomatic discs [5,6]. More broadly, systematic reviews have emphasized that suspected discogenic pain still lacks universally accepted diagnostic criteria and that noninvasive diagnostic methods remain imperfect [4].

For this reason, provocative discography has retained a role in selected patients despite longstanding controversy. Cloward originally described cervical discography as a means of linking disc stimulation to symptom reproduction [7]. Subsequent studies showed that cervical disc provocation can generate recognizable referral patterns and may help narrow the candidate levels in patients with multilevel degeneration [8]. A systematic review by Manchikanti et al. concluded that cervical discography, when performed according to accepted criteria, may be useful in evaluating chronic cervical pain in selected patients without radiculitis or frank disc herniation [9]. Nonetheless, discography is invasive, operator-dependent, and not ideal as a first-line diagnostic tool.

Ultrasound offers an appealing adjunct because it provides real-time imaging, portability, soft-tissue resolution, and freedom from ionizing radiation [10]. In cervical procedures, ultrasound is particularly advantageous because it allows direct visualization of vessels, viscera, nerves, muscular planes, and fascial interfaces that are not seen with fluoroscopy [10,11]. In this context, sonopalpation refers to the application of focused probe-mediated pressure during ultrasound examination to provoke symptoms while the underlying tissue response is visualized in real time. Lam et al. first described ultrasound-guided cervical intradiscal injection with fluoroscopic validation [12] and later reported a medial cervical approach in which direct digital sonopalpation was used to identify tender disc levels before injection [13]. Maeda et al. further showed that sonopalpation could help identify the most painful disc before ultrasound-guided cervical intradiscal injection without fluoroscopy [14]. More generally, the sonopalpation literature supports the value of probe-mediated mechanical provocation during ultrasound examination, although specific technical adaptations remain underreported [15].

A technical gap remains, however, in patients with challenging anterior neck anatomy. In some individuals with short necks, bulky anterior soft tissues, reduced tissue compliance, or close apposition of the carotid sheath to the thyroid-tracheal complex, conventional finger-based sonopalpation may be difficult or impossible even when the target disc can still be visualized sonographically. Although Lam et al. noted that a microconvex transducer can improve access to the medial cervical corridor [13], neither Lam et al. nor Maeda et al. described using the microconvex probe itself as the palpating instrument. This technical report describes a practical modification for such cases: use of the vertex of a small-footprint microconvex transducer as the sonopalpation instrument itself when conventional digital palpation is not feasible. In our preferred setup, the patient is seated and faces the ultrasound screen while the operator stands behind the patient, allowing real-time feedback during focused compression. This maneuver is intended as a problem-solving technical adaptation to extend the feasibility of ultrasound-guided disc level selection in difficult anatomy.

## Technical report

Concept and indication

**Figure 1:**
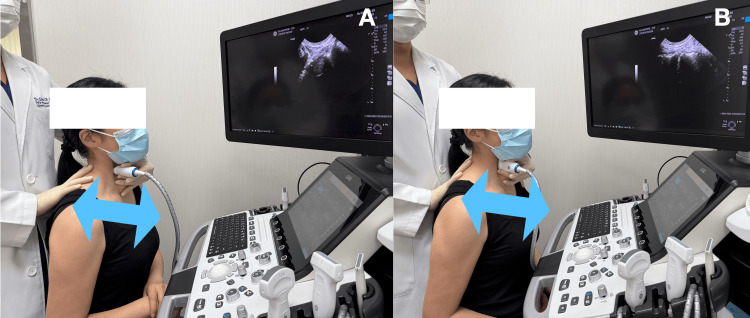
External positioning for microconvex probe-assisted cervical disc sonopalpation. (A) Short-axis probe orientation with the patient seated and facing the ultrasound screen while the operator stands behind the patient and applies the probe vertex for direct cervical disc compression. (B) Long-axis probe orientation used for level confirmation and alternative sonopalpation of the target disc. In the seated setup, gravitational loading of the head and cervical spine may help approximate the patient’s usual discogenic pain state and facilitate reproduction of concordant pain during focused probe-vertex sonopalpation. The double-headed arrows indicate the direction of the applied probe pressure. Image courtesy of Prof King Hei Stanley Lam.

The technique described here is intended for patients with suspected cervical discogenic pain in whom ultrasound-guided sonopalpation is desired but conventional digital palpation is limited by anterior neck anatomy. The specific problem addressed is a narrow or resistant interval between the carotid sheath laterally and the thyroid-tracheal complex medially, such that a finger or a broad linear transducer cannot create an adequate working corridor (Figures 1A, 1B).

The innovation is modest but clinically relevant: the microconvex transducer is used not only to visualize the target and create the corridor, but also to deliver the palpation maneuver itself. The goal is to reproduce the patient’s concordant pain by transmitting controlled focal pressure through the prevertebral musculature to the anterolateral annulus of the suspected disc under continuous ultrasound guidance.

Patient preparation and equipment

The examination is best performed in an awake, communicative patient so that concordant pain can be assessed in real time. In our preferred setup, the patient is seated facing the ultrasound screen while the operator stands behind and applies the microconvex transducer to the anterior cervical region (Figures 1A, 1B). This position permits continuous patient feedback and may help approximate the patient’s usual mechanical pain state through gravitational loading of the head and cervical spine. Under these circumstances, graded direct probe-vertex compression may more readily reproduce concordant pain.

Although we favor the seated setup when tolerated, patient positioning may be adapted according to operator preference, patient symptoms, and previously described anterior cervical ultrasound techniques [13,14], including semirecumbent or supine positioning when seated examination is not desirable or not well tolerated. Regardless of position, stable access to the anterior cervical soft tissues is required, and the carotid sheath, thyroid gland, trachea, prevertebral muscles, and target disc should be clearly visualized before provocative sonopalpation is attempted.

A small-footprint microconvex transducer is used, typically in the medium-frequency range suitable for anterior cervical imaging. The narrow curved footprint is the key technical feature: it allows the probe to enter a smaller interval than a standard linear transducer while still providing adequate penetration and field of view. Doppler imaging should be available and used when necessary to identify vascular structures.

Baseline ultrasound survey

**Figure 2:**
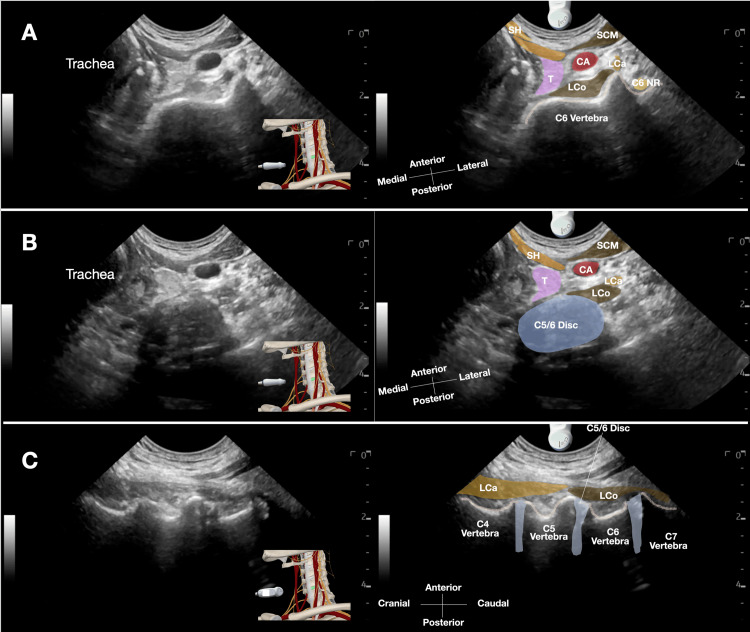
Ultrasound identification of the target cervical disc using short-axis and long-axis views. (A) Short-axis image at the C6 level, demonstrating the C6 vertebral body and C6 nerve root as segmental landmarks. (B) Short-axis image of the C5/6 disc, showing its relationship to the carotid artery, thyroid gland, trachea, and longus colli within the anterior cervical corridor. (C) Long-axis image demonstrating multilevel confirmation of the target C5/6 disc relative to the adjacent C4/5 and C6/7 disc levels and corresponding vertebral bodies. CA, carotid artery; C6 NR, C6 nerve root; LCa, longus capitis; LCo, longus colli; SH, sternohyoid; SCM, sternocleidomastoid; T, thyroid. Image courtesy of Prof. King Hei Stanley Lam

The target disc level is first identified using standard anterior cervical ultrasonography, with short-axis landmark recognition followed by long-axis confirmation across adjacent segments (Figures 2A-2C; Videos 1, 2). A transverse survey may be used initially to recognize cervical level landmarks, followed by confirmation in a longitudinal or oblique sagittal plane. Before any provocative maneuver is attempted, the operator should identify the carotid artery, internal jugular vein when relevant, thyroid gland, trachea, prevertebral fascia, longus colli, and the contour of the target disc (Figures 2A-2C).

**Video 1:** Short-axis identification of the cervical discs from C6/7 to C4/5. The transducer is used to obtain short-axis views of the lower cervical spine for landmark recognition and target disc localization while scanning from caudal to cranial, including confirmation of the C6/7, C5/6, and C4/5 discs and their relationships to the surrounding anterior cervical soft tissues. CA, carotid artery; NR, nerve root; LCa, longus capitis; LCo, longus colli; SH, sternohyoid; SCM, sternocleidomastoid; T, thyroid. VA, vertebral artery.

**Video 2:** Long-axis identification of the lower cervical discs from C6/7 to C4/5. After short-axis landmark recognition, the microconvex transducer is aligned with the long axis of the cervical spine to identify the C6 anterior tubercle as a segmental landmark. This facilitates longitudinal confirmation of the C6 vertebral body and the adjacent C6/7 and C5/6 disc levels, with the next cranial disc identified as C4/5. AT, anterior tubercle; LCa, longus capitis; LCo, longus colli

This baseline survey is essential because the procedure depends on precise spatial orientation rather than blind compression. If tissue planes are poorly visualized, probe-assisted sonopalpation should not be attempted.

Creation of the medial working corridor

Once the level is confirmed, the microconvex probe is advanced into the sagittal plane between the carotid sheath and the thyroid-tracheal complex (Video 3). Gentle, progressive pressure is used to separate these structures and create a narrow working corridor. In contrast to a flat linear probe, the curved, small-footprint microconvex transducer can wedge into this interval more readily, especially when soft tissues are tight or the neck is short. This step serves two functions simultaneously. First, it creates physical access to the prevertebral compartment. Second, it maintains real-time visualization of the surrounding anatomy while the corridor is being developed. The maneuver should remain slow and controlled, and it should be abandoned if tissue planes cannot be clearly resolved or if pressure produces diffuse superficial discomfort rather than focal deep provocation.

**Video 3:** Microconvex probe-assisted sonopalpation of the C5/6 cervical disc. The video begins with the microconvex probe aligned in the long axis of the cervical spine and the vertex positioned near the C4/5 disc. The probe is then slid caudally to the C5/6 level, where the target disc is shown at the probe vertex. The transducer is subsequently rotated 90° to obtain a short-axis view, increasing contact between the probe vertex and the anterolateral aspect of the C5/6 disc. Direct probe-vertex sonopalpation is then applied, and the probe may be alternated between long-axis and short-axis orientations to assess reproduction of concordant pain. CA, carotid artery; NR, nerve root; LCa, longus capitis; LCo, longus colli; LF, ligamentum flavum; SH, sternohyoid; SCM, sternocleidomastoid; T, thyroid; OMH, omohyoid.

Probe-vertex sonopalpation

**Figure 3:**
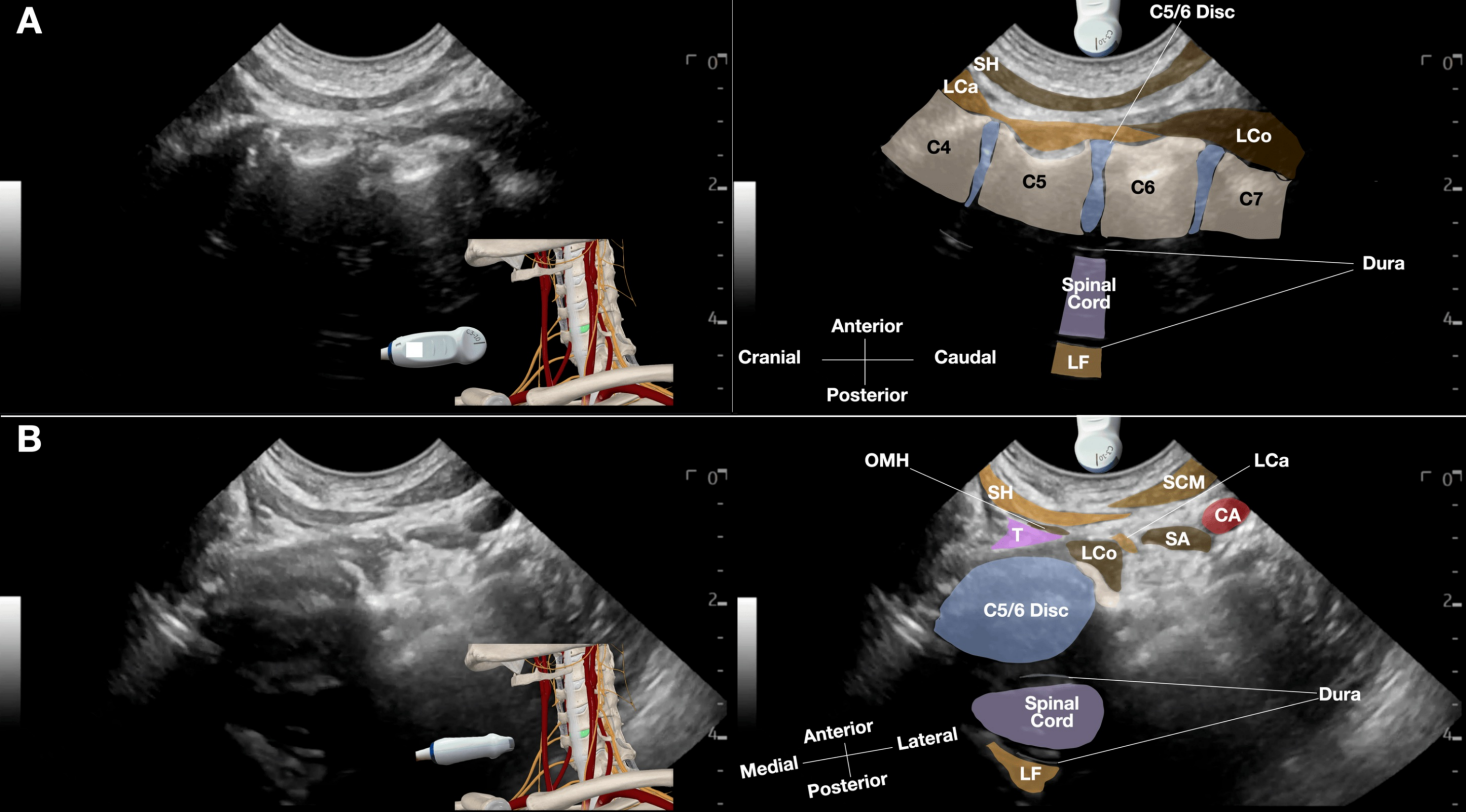
Ultrasound still images demonstrating microconvex probe-assisted sonopalpation of the C5/6 disc. (A) Long-axis sonopalpation of the C5/6 disc using the probe vertex. (B) Short-axis sonopalpation of the C5/6 disc, showing broader probe contact with the anterolateral disc region during focused compression. Direct probe-vertex sonopalpation is applied to the anterolateral C5/6 disc while the carotid artery is displaced laterally. CA, carotid artery; NR, nerve root; LCa, longus capitis; LCo, longus colli; LF, ligamentum flavum; SH, sternohyoid; SCM, sternocleidomastoid; T, thyroid; OMH, omohyoid. Image courtesy of Prof. King Hei Stanley Lam.

After the corridor is established, the operator uses the vertex of the microconvex transducer as the palpating surface (Figures 3A, 3B; Video 3). The transducer may initially be aligned in the long axis of the cervical spine and slid to the target level, then rotated 90° to obtain a short-axis view that increases the contact area between the probe vertex and the anterolateral aspect of the target disc (Figures 1A, 1B; Figures 3A, 3B; Video 3). The probe may be alternated between long-axis and short-axis orientations according to which view provides better contact, stability, and visualization during sonopalpation.

The transducer is subtly pivoted so that focal pressure is concentrated through the prevertebral fascia and longus colli toward the anterolateral annulus fibrosus of the target disc. At more cephalad levels, pressure may also be transmitted through the longus capitis as anatomically appropriate. When performed in the seated position, the natural gravitational load of the head and cervical spine may help approximate the patient’s usual mechanical pain state, which may facilitate reproduction of concordant symptoms during focused probe-vertex sonopalpation.

Compression is applied in a graded fashion rather than abruptly. The purpose is not to produce generalized discomfort, but to mechanically stress the suspected disc level while directly observing tissue displacement. During this maneuver, the patient is asked whether the provoked pain reproduces the usual symptoms in quality and location. Reproduction of concordant pain is taken as a clinically useful sonopalpation finding, particularly in the setting of multilevel degenerative change on MRI. In the illustrative application described in this report, concordant pain was reproduced without immediate procedure-related adverse events.

Multi-level assessment

If several discs appear potentially symptomatic on clinical or imaging grounds, the same maneuver may be repeated at adjacent levels. The level or levels that reproduce the patient’s typical pain pattern most clearly may then be prioritized for subsequent diagnostic or therapeutic intervention, consistent with the previously described sonopalpation-based approach [13,14].

Transition to intervention

If an intradiscal procedure is planned, the same microconvex transducer may continue to be used for ultrasound-guided disc access according to previously described medial cervical approaches [13,14]. The present report does not propose a new needle trajectory. Rather, it adds a specific sonopalpation modification that may improve pre-procedural level selection when digital palpation cannot be performed effectively.

Practical advantages and limitations

The proposed advantages of this technique arise from the physical properties of the microconvex probe. Its narrow footprint facilitates entry into a constrained anterior cervical corridor; its curved apex acts as a blunt separator; and its vertex can transmit focal pressure while maintaining imaging continuity. The seated setup may provide an additional practical advantage, as gravitational loading of the head and cervical spine may help approximate the patient’s usual symptomatic state during the examination. The main limitation is loss of tactile feedback compared with the examining finger. The operator must rely more heavily on ultrasound visualization, controlled pressure application, and the patient’s report of concordant pain. Table 1 shows practical comparison between conventional digital sonopalpation and microconvex probe-assisted sonopalpation for evaluation of suspected cervical discogenic pain.

**Table 1:** Practical comparison between conventional digital sonopalpation and microconvex probe-assisted sonopalpation for evaluation of suspected cervical discogenic pain. This table summarizes the main technical differences between the two approaches, including the palpating surface, method of corridor creation, imaging integration, suitability for challenging anterior neck anatomy, tactile feedback, and principal advantages and limitations.

Feature	Conventional digital sonopalpation	Microconvex probe-assisted sonopalpation
Palpating surface	Finger	Probe vertex
Corridor creation	Finger and/or probe	Probe alone
Visualization during palpation	Separate imaging and palpation roles	Integrated imaging and palpation
Suitability for short, tight neck anatomy	May be limited	Potentially favorable
Tactile feedback	Excellent	Reduced
Main technical advantage	Rich haptic information	Access and continuous visualization
Main technical limitation	Finger may not fit into corridor	Less haptic discrimination

## Discussion

This report describes a technical extension of previously published ultrasound-guided cervical disc diagnostic methods rather than an entirely new conceptual framework. Lam et al. and Maeda et al. established the practical basis for ultrasound-guided sonopalpation in suspected cervical discogenic pain by showing that mechanical provocation under real-time imaging may help identify painful cervical disc levels before intradiscal intervention [13,14]. The present contribution is narrower but potentially useful: in anatomically constrained patients, the microconvex transducer vertex can serve as the sonopalpation instrument itself, rather than functioning only as an imaging device or corridor-opening probe.

That distinction is clinically meaningful because cervical discogenic pain remains a diagnostic gray zone. Cervical discs are biologically capable of generating pain, yet the clinical evidence base is still less definitive than many clinicians would prefer [2-4]. MRI is valuable for structural assessment, but it is not a reliable stand-alone test for identifying the symptomatic disc [4-6]. Discography remains the best-known method for linking a specific disc to concordant pain reproduction, but it is invasive and controversial [7-9]. In routine practice, any maneuver that improves level selection while preserving anatomical visualization is therefore attractive, especially when multiple cervical discs show degeneration on MRI.

Sonopalpation is appealing because ultrasonography is uniquely suited to dynamic symptom provocation. Unlike static imaging, ultrasound allows the operator to mechanically challenge a suspected structure while simultaneously observing the tissue plane being stressed [15]. In the anterior cervical region, this dynamic capability is especially useful because the operator is working within a crowded corridor bounded by the carotid sheath, thyroid gland, trachea, prevertebral fascia, and longus muscles [10,11,16]. Real-time visualization may improve confidence that pressure is being transmitted to the intended prevertebral muscular layer and annular region rather than to more superficial or adjacent structures.

The present modification is best understood as a problem-solving technique for difficult anatomy. In patients with short necks, thick anterior soft tissues, reduced tissue compliance, or minimal separation between the carotid sheath and the thyroid-tracheal complex, finger-based sonopalpation may be impractical because the examining finger cannot enter the corridor effectively. A standard linear transducer may be similarly limited by its broader footprint. By contrast, the small curved apex of the microconvex probe can help create the corridor and then deliver focal, graded compression to the prevertebral muscles and underlying annulus under continuous imaging. Features that complicate conventional digital palpation may therefore define the subgroup most likely to benefit from this approach.

The seated setup may help approximate the patient’s usual symptomatic condition during focused compression, although this should be understood as a procedural rationale rather than a proven comparative advantage. This point may nevertheless be clinically relevant when attempting to reproduce mechanically sensitive disc pain in a setting that more closely resembles the patient’s usual upright symptomatic state.

This also sharpens the novelty claim. Lam et al. had already recognized that a microconvex transducer can facilitate exposure of the medial cervical corridor [13]. Accordingly, the novelty of the present report should not be framed as the mere use of a microconvex probe in cervical disc procedures. Rather, the technical novelty lies in using the probe vertex to replace the examining finger for sonopalpation when direct digital palpation is difficult or impossible. This more precise framing is both more accurate and more defensible.

Several practical advantages follow from this modification. First, one instrument can create the working corridor, visualize the target, and deliver the provocative maneuver. Second, continuous imaging may help the operator monitor the relationship between the probe, the carotid sheath, the thyroid-tracheal complex, and the prevertebral tissues throughout the examination. Third, the method may expand the feasibility of sonopalpation in patients who would otherwise be poor candidates for conventional digital palpation. These are plausible technical advantages, not proven comparative outcomes, and they should be presented as such.

The main trade-off is reduced tactile feedback. A plastic transducer tip cannot reproduce the nuanced haptic information provided by the examining finger. Subtle differences in tissue texture, depth resistance, and compliance are less perceptible, making the maneuver more visually driven and potentially more operator-dependent. In addition, the pressure applied through the probe must be carefully titrated; excessive force may produce nonspecific soft-tissue discomfort rather than meaningful concordant pain. For these reasons, the technique should be considered a selective adjunct rather than a universal replacement for digital sonopalpation.

Safety considerations are central. The anterior cervical corridor contains critical vascular and visceral structures, and the sympathetic trunk lies anterior to the longus muscles beneath the prevertebral fascia [17]. A clear understanding of infrahyoid and perithyroidal sonoanatomy is therefore essential before attempting this maneuver [11,16]. Continuous visualization should be maintained throughout, and Doppler should be used when necessary to identify vascular structures. If the operator cannot clearly resolve the tissue planes, the maneuver should not be performed. Likewise, abrupt or forceful compression should be avoided.

The limitations of the present report should be stated plainly. This is a technical description, not a diagnostic accuracy study. It does not establish sensitivity, specificity, inter-rater reliability, comparative tolerability, or superiority over conventional digital sonopalpation. It also does not prove that concordant pain reproduced by probe-vertex compression is diagnostically equivalent to concordant pain elicited by formal discography. Although concordant pain was reproduced without immediate procedure-related adverse events in the illustrative application described here, this observation is anecdotal and should not be interpreted as proof of diagnostic validity or procedural safety. Those questions require prospective study. At present, the most appropriate conclusion is that the method appears technically feasible and logically suited to selected patients with challenging anterior neck anatomy.

A hybrid variation may also be possible. In patients with partially accessible anatomy, the microconvex probe may be used to create the working corridor while adjunctive digital palpation provides additional tactile feedback. However, this hybrid approach is not the focus of the present report and has not been systematically evaluated. Mentioning it as a potential procedural variant is reasonable, but it should not dilute the manuscript’s central claim.

## Conclusions

Microconvex probe-assisted sonopalpation is a practical technical modification for ultrasound-guided evaluation of suspected cervical discogenic pain when conventional digital palpation is limited by challenging anterior neck anatomy. The key innovation is not the use of a microconvex transducer for visualization alone, but the use of its vertex as the palpating instrument itself under continuous ultrasound guidance.

This approach may extend the feasibility of cervical disc sonopalpation in patients with short necks, bulky anterior soft tissues, or a tightly constrained medial cervical corridor. In addition, seated positioning may help approximate the patient’s usual symptomatic condition through gravitational loading of the head and cervical spine during focused compression. Its principal limitation remains reduced tactile feedback compared with finger-based palpation. At present, this method should be regarded as a selective, anatomy-driven adjunct rather than a validated replacement for established diagnostic strategies. Further study is warranted to define reproducibility, safety, and diagnostic performance.
